# Strain-dependent duodenal structural alterations in hamsters infected with *Leishmania (Viannia) braziliensis*

**DOI:** 10.1590/S1678-9946202668038

**Published:** 2026-06-12

**Authors:** Maria Gabriela Lima da Silva-Zussa, Amanda Gubert Alves dos Santos, Thaís Gomes Verzignassi Silveira, Andrea Claudia Bekner Silva Fernandes, Lainy Leiny de Lima, Roberto Keiji Nakamura Cuman, Débora de Mello Gonçales Sant’Ana, Gessilda de Alcantara Nogueira-Melo

**Affiliations:** 1 Universidade Estadual de Maringá, Programa de Pós-Graduação em Biociências e Fisiopatologia, Maringá, Paraná, Brazil; 2 Universidade Estadual de Maringá, Programa de Pós-Graduação em Ciências da Saúde, Maringá, Paraná, Brazil; 3 Universidade Estadual de Maringá, Departamento de Análises Clínicas e Biomedicina, Maringá, Paraná, Brazil; 4 Universidade Paranaense, Programa de Pós-Graduação em Ciência Animal com Ênfase em Produtos Bioativos, Umuarama, Paraná, Brazil

**Keywords:** Cutaneous leishmaniasis, Small intestine, Histology

## Abstract

*Leishmania (Viannia) braziliensis* (LVB), the main etiological agent of cutaneous and mucocutaneous leishmaniasis, may cause atypical manifestations in immunosuppressed hosts, affecting organs beyond the skin, including the intestine. This study investigated whether the duodenum is involved during experimental LVB infection in hamsters. Overall, 48 female golden hamsters were divided into a control and infected group with five LVB strains. They were euthanized after 90 or 120 days of infection. Duodenal samples were subjected to histological and morphometric analyses, including evaluation of the duodenal wall, villi dimensions, intraepithelial lymphocytes, goblet cells, mast cells, collagen fiber composition (types I and III), and myenteric ganglia. Biochemical assays were performed to assess myeloperoxidase activity and nitrite levels. LVB infection reduced Alcian Blue pH 1.0–positive goblet cells and myenteric ganglia area at 90 days and increased type III collagen fibers at 120 days. Infected animals showed increased villus width at 90 days and greater villus height at 120 days. Overall, LVB infection induces significant and time-dependent alterations in the duodenal histoarchitecture, including epithelial, neuronal, and extracellular matrix changes, indicating that the duodenum undergoes dynamic structural remodeling during the infection.

## INTRODUCTION

Leishmaniases are parasitic diseases caused by *Leishmania* protozoa, transmitted primarily via the bite of infected female phlebotomine sandflies during blood feeding^1^. Their clinical outcome and severity depend on the species or strain, resulting in distinct clinical cutaneous, mucocutaneous, and visceral leishmaniasis manifestations^2^. Over the past two decades, the Pan American Health Organization has reported an annual average of 53,387 cutaneous and mucocutaneous cases^3^. Notably, in some instances, species typically associated with cutaneous or mucocutaneous forms can also lead to visceral involvement^4^.

Brazil has several *Leishmania* species, among which *L. (V.) braziliensis* is particularly noteworthy as the first species to be described as the etiological agent of tegumentary leishmaniasis in the Americas. Currently, this species occurs in all Brazilian states, causing most cases nationwide^5^. *L. (V.) braziliensis* shows marked genetic polymorphism^6^, variable infectivity and virulence, and considerable differences in responsiveness to anti-*Leishmania* therapy^6,7^. Genetically distinct strains may coexist within a single geographic area, resulting in diverse clinical outcomes^8,9^ and therapeutic responses, even among patients undergoing similar treatments^10,11^.

Interestingly, studies have shown that *Leishmania* parasites can disseminate to extra-cutaneous sites, including the blood, bone marrow^8^, spleen^9,10^, liver^10,11^, and the intestines^12^. Intestinal infection by *Leishmania* has been documented in rodents^13,14^ and dogs^15^. Among experimental models, hamsters have proven to be particularly suitable as their clinical, histopathological, and immunological features closely resemble that of human infection^9,13,16^.

Santos *et al*.^12,13^ have reported cellular, morphological, and neuronal alterations in the ileum of female hamsters infected with *L. (V.) braziliensis*. Building on these findings, this study aims to determine whether the duodenum of female hamsters also shows histopathological and biochemical changes during infection with *L. (V.) braziliensis* at 90 and 120 days post-infection.

This study investigates whether infection with several *L. (V.) braziliensis* strains induces histomorphological and biochemical alterations in the duodenum of hamsters. We evaluated intestinal morphometry, goblet cell mucin production, immune cell infiltration, collagen fiber composition, inflammatory mediators, and myenteric ganglion morphology at two infection times (90 and 120 days).

## MATERIALS AND METHODS

### Ethics and animal model

All experimental procedures were approved by the Animal Ethics Committee of Universidade Estadual de Maringa under protocol 7587260416. A total of 48 female golden hamsters (*Mesocricetus auratus*) aged 12 weeks were obtained from the Central Animal Facility of Universidade Estadual de Maringa. Animals were randomly assigned into six groups (n = 8): one control and five infected groups. Each group was subdivided into two time points, 90 or 120 days post-infection, resulting in 12 experimental groups (n = 4 each).

### Experimental design

The reference strain *Leishmania (V.) braziliensis* MHOM/BR/1975/M2903 (2903) and four additional clinical isolates obtained from patients treated at Laboratorio de Ensino e Pesquisa em Analises Clinicas, Universidade Estadual de Maringa, Maringa, Parana State, Brazil (−23.402168801897535, −51.94109661352054) were used. Clinical outcomes determined the classification of isolates: MHOM/BR/2003/2314 (2314) was treatment-sensitive, with complete lesion regression after therapy; MHOM/BR/2003/2311 (2311) was moderately resistant as lesion cure occurred only after a second treatment course; MHOM/BR/2000/1655 (1655) caused lesion reactivation after apparent cure; and MHOM/BR/2009/3476 (3476) induced atypical lesions and showed poor therapeutic response. Parasites were cultured, cryopreserved, and identified at the Oswaldo Cruz Institute, Rio de Janeiro, Brazil^17^.

Promastigotes were thawed and cultured in medium 199 (Gibco Laboratories^®^, Grand Island, USA) supplemented with 1% L-glutamine, 1% human urine, and 10% fetal bovine serum. Animals were anesthetized with 10 mg/kg of xylazine (Calmiun^®^, Agener-Union Animal Health) and 50 mg/kg of ketamine (Francotar^®^, Virbac Animal Health) and intradermally inoculated in the dorsal surface of their left hind paw with 100 μL containing 2 × 10^7^ promastigotes. Control animals received 100 μL of phosphate-buffered saline under identical conditions.

### Duodenum sampling

At each experimental endpoint, animals were euthanized under deep anesthesia. A 1-cm segment of the duodenum was collected and fixed in 4% buffered paraformaldehyde for histological processing. An additional 0.5-cm fragment was reserved for biochemical assays.

### Histological processing

Fixed samples were dehydrated, cleared, and embedded in paraffin. Semi-serial 5-μm transverse sections were stained with hematoxylin and eosin for the quantification of intraepithelial lymphocytes (IELs) and the morphometric analysis of intestinal wall, villi, crypts, and myenteric ganglia. Histochemical methods included periodic acid-Schiff for neutral mucin-producing goblet cells; Alcian blue at pH 2.5 and pH 1.0 for sialomucin- and sulfomucin-producing goblet cells, respectively; picrosirius red was used for collagen fiber assessment; and toluidine blue, for mast cell quantification.

### Quantification of goblet cells, IELs, and mast cells

Each section was divided into four quadrants, and 160 epithelial cells per quadrant (totaling 2,560 per animal) were counted for goblet cells and IELs. Results are shown as ratios per 100 epithelial cells^18,19^. Mast cells were quantified in toluidine blue-stained sections by counting all cells in 100 random microscopic fields (100× objective) from four sections per animal. Results are shown as mast cells per mm^2 17,18^.

### Intestinal wall measurements

Morphometric measurements were performed on Motic Images Plus 2.0 (version 2.0, Motic China Group Co., Ltd., Hong Kong, China). In total, 16 images per animal (four sections, one image per quadrant) were acquired with a digital camera (Moticam 2000, 2.0 MP) attached to a trinocular light microscope (MOTIC B5). Using 10× magnification, villus height and width, crypt depth and width, submucosal thickness, muscle layers, and total duodenal wall thickness were determined, totaling 64 measurements per parameter per animal^17,18^.

### Collagen fiber analysis

Picrosirius red-stained sections were analyzed for type I and type III collagen under polarized light using an Olympus BX50 microscope (Minato-Ku, Japan). In total, 16 images per animal (one per quadrant per section) were acquired at 20× magnification. Additional non-polarized images were captured to assess total collagen content. Fiber area (μm^2^) was quantified on Image-Pro^®^ Plus (versio 4.5.0.29, Media Cybernetics, MD, USA)^18^.

### Biochemical analyses: Myeloperoxidase activity and nitrite quantification

Myeloperoxidase activity was determined using the homogenate supernatant from each small duodenal segment (50 mg). To obtain the homogenate, a 50-mM phosphate buffer solution (pH 6.0) containing 0.5% trimethyl hexadecyl ammonium bromide (1 mL/50 mg of tissue; Sigma^®^) was added to the samples in the homogenizer. Then, 10 μL of the supernatant were added in duplicate to a 96-well microplate. Subsequently, 200 μL of the substrate solution (16.7 mg of o-dianisidine dihydrochloride, Sigma^®^; 90 mL of double-distilled water; 10 mL of phosphate buffer; and 50 μL of 1% H_2_O_2_) were added. The enzymatic reaction was stopped by adding 30 μL of sodium acetate (Sigma^®^). Optical density was measured at 460 nm (Asys Expert Plus^®^). The results are shown as optical density^20^.

Nitrate levels were determined using the Griess method, which defines nitrite production as a measure of NO production^21^. In triplicates and at room temperature, 50 μL of the small intestine segment homogenate was pipetted into a 96-well microplate, to which the Griess reagent was added (1% sulfanilamide in 5% phosphoric acid and 0.1% N-(1-naphthyl) ethylenediamine in water, Sigma^®^). Then, absorbance was measured at 550 nm (Asys Expert Plus^®^)^22^. NO concentrations were calculated from a sodium nitrite standard curve. The results are shown in μM^23^.

### Histomorphometric analysis of myenteric ganglia

The area of 10 myenteric ganglia of each animal was evaluated in hematoxylin and eosin-stained sections of the duodenum. Images were captured using a 40x objective, and the mean area per animal was calculated for statistical analysis. The results are shown as group means in μm^19^.

### Statistical analysis

Statistical analyses were performed on GraphPad Prism (version 8.0.1, GraphPad Software, Inc. San Diego, California, EUA). Normality was assessed by the Shapiro–Wilk test. All data were normally distributed. Two-way ANOVA followed by Tukey's post hoc test was applied for group comparisons. Significance was set at p < 0.05 (95% confidence interval). Results are shown as mean ± standard deviation (SD).

## RESULTS

### Clinical evaluation

**Figure 1:**
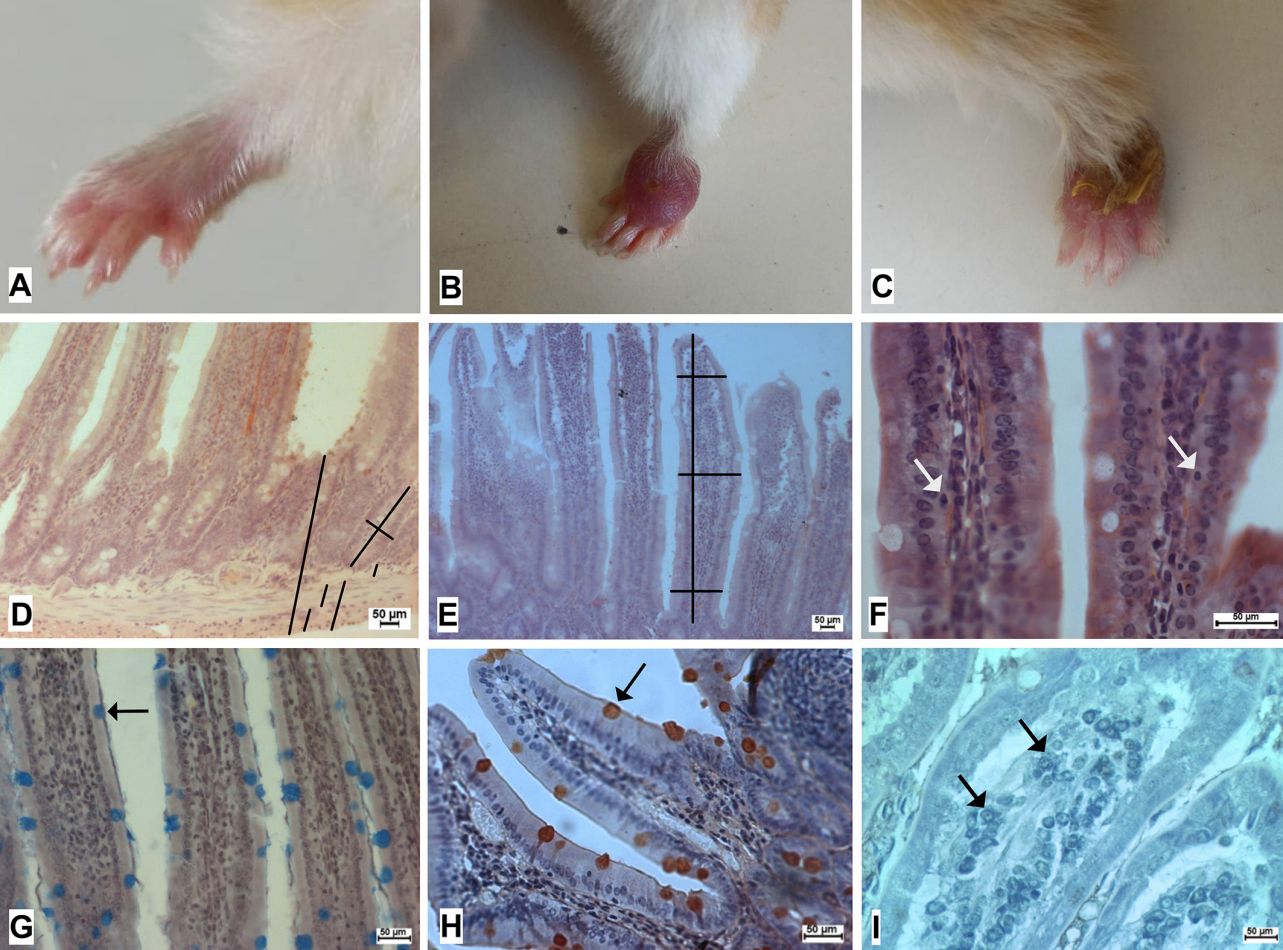
Representative photographs of the changes caused by L. (V.) braziliensis infection in the paws of infected hamsters, showing the development of lesions after infection: (A) control group, (B) edema and initial lesion, (C) ulcerated lesion. Schematic representation of the measurement of (D) total wall, muscular layers, submucosa depth, and width of the crypts, respectively, and (E) height and width of the villi. A total of 64 measurements of each parameter were performed for each animal. To obtain the width of the villi, the base, middle, and apex were measured. (F) Representative photomicrograph of intraepithelial lymphocytes (IELs). Goblet cells in villi stained with Alcian blue pH 1.0 (G) and periodic acid-Schiff (H); (I) Representative photomicrograph of mast cells.

These animals showed no overt systemic clinical alterations, such as changes in body weight, coat condition, or stool characteristics according to published data^12,13^. This study confirmed the infection by the presence of progressive cutaneous lesions at the site of parasite inoculation, observed in all animals throughout the experimental period^12,13^. The progression of these lesions were associated with mild impairment of mobility (Figures 1A to AC).

### Duodenal morphometry

**Figure 2:**
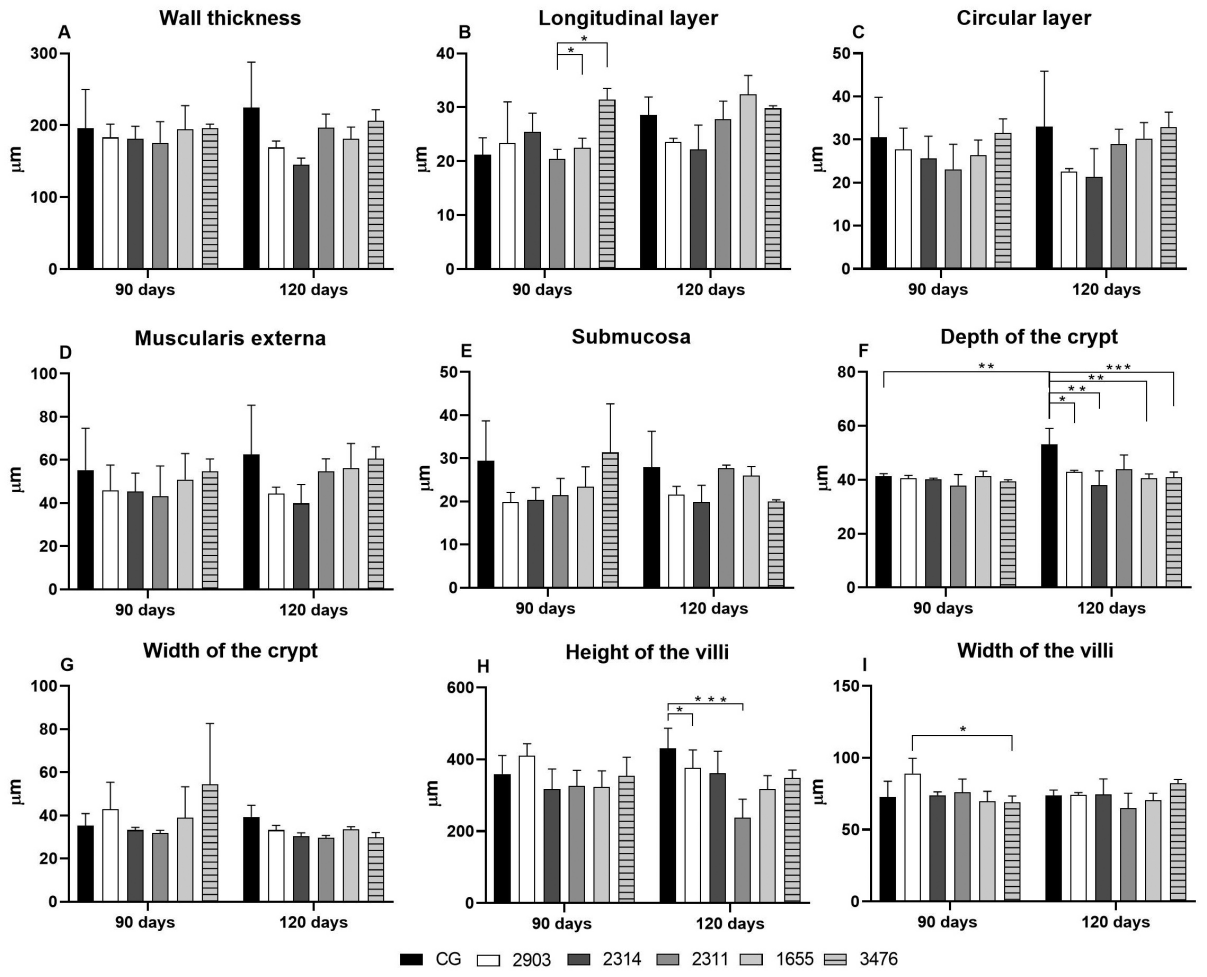
Morphological alterations in duodenal villi of female hamsters infected with different Leishmania (V.) braziliensis strains at 90 and 120 days post-infection. Groups: CG = non-infected control; 2314 = infection with strain 2314 (treatment-sensitive); 2311 = infection with strain 2311 (moderately resistant); 1655 = infection with strain 1655 (reactivation after cure); 3476 = infection with strain 3476 (atypical lesions). Data are shown as mean ± SD (n = 4 per group). *p < 0.05, **p < 0.01, ***p < 0.001 (two-way ANOVA with Tukey's post hoc test).

This study observed no macroscopic alterations in duodenal length, color, or gross structure, with an overall mean length of 5.01 ± 0.63 cm across groups. Figure 2 represents duodenal morphometry. Histological analysis showed a significant reduction in crypt depth at 120 days post-infection in animals infected with strains 2903, 2314, 1655, and 3476 when compared to their respective controls, indicating an infection effect [F_(5,24)_ = 4.75; *p* = 0.0037]. Interestingly, this study also found an age-related increase in crypt depth in control animals at 120 days when compared to controls at 90 days [F_(1,54)_ = 8.56; *p* = 0.074] (Figure 2F).

At 90 days, infection with strain 2903 was associated with a greater villus width than that of strain 3476 [F_(5,33)_ = 3.341; *p* = 0.0150] (Figure 2I). At 120 days, villus height decreased in the group infected with strain 2311 when compared to the control [F_(5,33)_ = 6.236; *p* = 0.0004] (Figure 2H). This study observed no significant differences in total wall, longitudinal and circular muscle, or submucosa thickness across groups (Figures 2A to 2E).

### Goblet cells and mucin secretion

**Figure 3:**
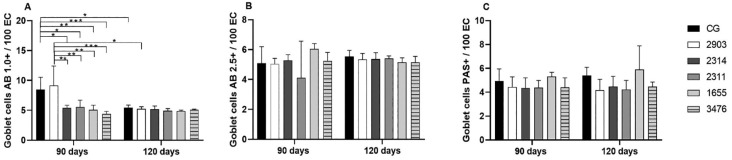
Goblet cell counts in the duodenal mucosa of female hamsters infected with Leishmania (V.) braziliensis at 90 and 120 days post-infection. Alcian blue staining showed reduced sulfomucin-producing goblet cells after infection. Quantitative analysis is shown as mean ± SEM (n = 4). One-way ANOVA with Tukey's post hoc test was used for comparisons. Groups: CG = non-infected control; 2314 = infection with strain 2314 (treatment-sensitive); 2311 = infection with strain 2311 (moderately resistant); 1655 = infection with strain 1655 (reactivation after cure); 3476 = infection with strain 3476 (atypical lesions). Data are shown as mean ± SD (n = 4 per group). *p < 0.05, **p < 0.01, ***p < 0.001 (two-way ANOVA with Tukey's post hoc test).

This study observed a significant reduction in the proportion of Alcian Blue pH 1.0-positive goblet cells in 120-day-old control animals when compared with the 90-day-old controls [F_(1,33)_ = 12.78; *p* = 0.0011], indicating an age-related effect (Figure 3A). Infection further decreased the proportion of acidic mucin-secreting goblet cells at 90 days, particularly in animals infected with strains 2314, 2311, 1655, and 3476, when compared to control and strain 2903 groups [F_(5,33)_ = 6.475; *p* = 0.0003], with a significant interaction effect [F_(5,33)_ = 4.609; *p* = 0.0027]. No significant differences were observed in the proportion of goblet cells stained with Alcian Blue pH 2.5 or periodic acid-Schiff at either infection time (Figures 3Bf and 3C).

### Intraepithelial lymphocytes, mast cells, and collagen fiber composition

**Figure 4:**
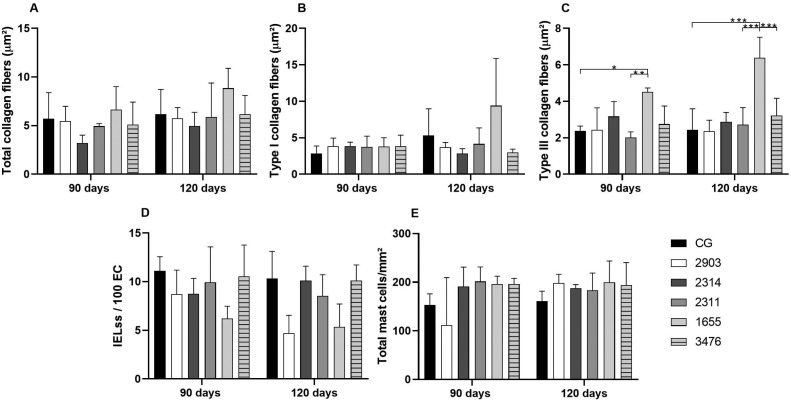
Collagen deposition in the duodenal submucosa of female hamsters infected with Leishmania (V.) braziliensis at 90 and 120 days post-infection. Picrosirius red staining was used to assess collagen fiber area: (A) total collagen fibers, (B) type I collagen fibers, (C) type III collagen fibers, (D) IELs, and (E) total mast cells. Data are shown as mean ± SEM (n = 4). One-way ANOVA followed by Tukey's post hoc test. Groups: CG = non-infected control; 2314 = infection with strain 2314 (treatment-sensitive); 2311 = infection with strain 2311 (moderately resistant); 1655 = infection with strain 1655 (reactivation after cure); 3476 = infection with strain 3476 (atypical lesions). Data are shown as mean ± SD (n = 4 per group). *p < 0.05, **p < 0.01, ***p < 0.001 (two-way ANOVA with Tukey's post hoc test).

This study observed no significant alterations in total collagen content (Figure 4A) or in type I collagen fibers (Figure 4B) across infection groups and time points. In contrast, type III collagen fibers (Figure 4C) showed a significant increase in animals infected with strain 1655 when compared with the control group at 90 days post-infection. This increase persisted at 120 days and was also higher than that observed in animals infected with strains 2311 and 3476. Representative photomicrographs under polarized light illustrate the enhanced deposition of type III collagen fibers in the duodenum of 1655-infected hamsters.

No significant differences were detected in the number of IELs (Figure 4D) or total mast cells in the duodenum of infected hamsters when compared with their respective controls, regardless of parasite strain or infection duration (Figure 4E). These findings indicate that *L. (V.) braziliensis* infection fails to significantly affect immune cell infiltration within the duodenal epithelium or lamina propria under the evaluated experimental conditions.

### Nitric oxide and myeloperoxidase activity

**Figure 5:**
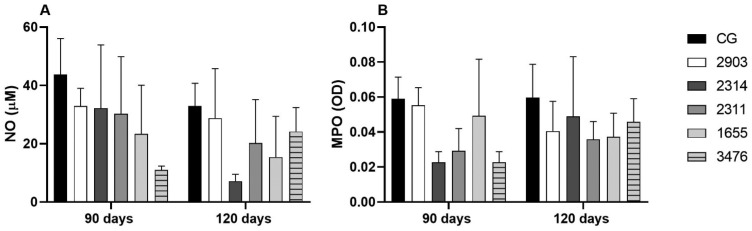
(A) Nitrite (NO) and (B) myeloperoxidase (MPO) activity levels in the duodenum of female hamsters infected with Leishmania (V.) braziliensis at 90 and 120 days post-infection. Results are shown as mean ± SEM (n = 4 animals per group). Statistical significance was assessed by one-way ANOVA followed by Tukey's post hoc test. Groups: CG = non-infected control; 2314 = infection with strain 2314 (treatment-sensitive); 2311 = infection with strain 2311 (moderately resistant); 1655 = infection with strain 1655 (reactivation after cure); 3476 = infection with strain 3476 (atypical lesions). Data are shown as mean ± SD (n = 4 per group). *p < 0.05, **p < 0.01, ***p < 0.001 (two-way ANOVA with Tukey's post hoc test).

This study observed no significant differences in nitrite levels (Figure 5A) or myeloperoxidase activity (Figure 5B) in the duodenum of infected hamsters when compared with their respective controls, regardless of parasite strain or infection duration. These findings suggest that *L. (V.) braziliensis* infection fails to substantially change local nitric oxide production or neutrophil-related enzymatic activity under the evaluated experimental conditions.

### Myenteric ganglion morphometry

**Figure 6:**
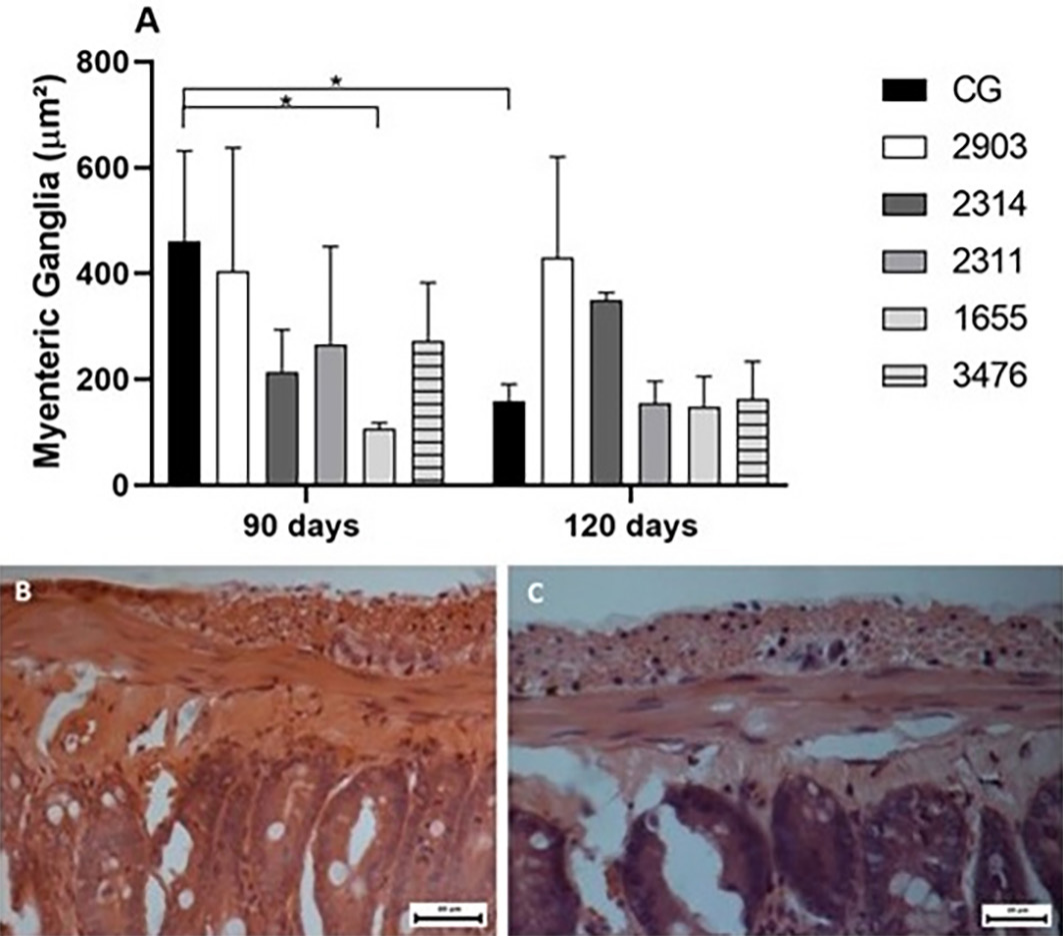
Myenteric plexus alterations in female hamsters infected with Leishmania (V.) braziliensis at 90 and 120 days post-infection. Representative micrographs and morphometric quantification of neuronal ganglion area are shown. Results are expressed as mean ± SEM (n = 4 animals per group). Statistical significance was assessed by one-way ANOVA followed by Tukey's post hoc test. Groups: CG = non-infected control; 2314 = infection with strain 2314 (treatment-sensitive); 2311 = infection with strain 2311 (moderately resistant); 1655 = infection with strain 1655 (reactivation after cure); 3476 = infection with strain 3476 (atypical lesions). Data are shown as mean ± SD (n = 4 per group). *p < 0.05, **p < 0.01, ***p < 0.001 (two-way ANOVA with Tukey's post hoc test).

This study observed a significant reduction in the mean area of myenteric ganglia in animals infected with strain 1655 when compared with the control group at 90 days post-infection (Figure 6A). It also detected an age-related effect as the control animals at 120 days showed smaller ganglion areas than those at 90 days (*p* < 0.05). Representative photomicrographs illustrate the structural organization of myenteric ganglia in control (Figure 6B) and strain 1655-infected animals (Figure 6C).

## DISCUSSION

Although *L. (V.) braziliensis* infection is classically associated with cutaneous and mucocutaneous lesions, previous studies have shown parasite dissemination to visceral organs such as the liver, spleen^11,13^, and intestines^14^. Based on this evidence, we investigated whether infection with distinct *L. (V.) braziliensis* strains induces cellular and structural changes in the duodenum of female hamsters after 90 and 120 days of infection.

The small intestinal villi are lined by a highly specialized epithelial cell layer responsible for nutrient digestion and absorption while also acting as a first line of defense against luminal antigens and pathogens. These epithelial cells arise from stem cells within the intestinal crypts and contribute to innate immunity by forming a physical and biochemical barrier^24,25^. This study observed a reduction in sulfomucin-producing goblet cells, which are critical for maintaining mucus production. Mucins lubricate the gastrointestinal surface and protect the epithelium from gastric acid and digestive enzymes, preventing microbial and antigenic penetration and maintaining intestinal immune microbiota homeostasis^26-29^. The dysfunction of goblet cells has been associated with increased intestinal permeability, dysbiosis, and altered mucosal immunity^30,31^. Notably, damage to goblet cells appears to be a common finding in animals infected with *Leishmania* spp. A previous report observed a decrease of goblet cells in the jejunum in animals infected with *L. (L.) infantum* ^30^. The damage to goblet cells in the mucosa likely results from inflammatory responses and an exacerbated immune response, which might lead to progressive tissue destruction in oropharyngeal mucosal lesions. Interestingly, the literature has reported no significant alterations in goblet cell numbers in the ileum of hamsters infected with the same *L. (V.) braziliensis* strains^12,13^, suggesting that the intestinal segments may respond differentially to the infection.

In addition to changes in mucus-secreting cells, we observed reduced villus height and width and crypt depth at 120 days, particularly in animals infected with strains 2903 and 1655. These findings disagree with Santos *et al*.^12,13^, who reported increased villus size and epithelial proliferation in the ileum following infection. Decreased crypt depth is often associated with reduced epithelial cell proliferation^31^, which could impair the production and maturation of specialized epithelial cells (including goblet cells) and explain the shortened villi in this study.

We also detected increased type III collagen deposition in the submucosa, mainly in animals infected with strain 1655. Collagen remodeling is mediated by a complex network of cytokines, adhesion molecules, and growth factors, increasing extracellular matrix deposition^32,33^. Collagen type III plays an important structural role, supporting tissue repair and local architecture during inflammatory or remodeling processes^34^. Our results suggest that chronic *Leishmania* infection, even when primarily cutaneous, can trigger extracellular matrix remodeling in distal tissues.

The enteric nervous system (ENS) also seemed affected, as evinced by the reduced myenteric ganglion area in animals infected with strain 1655 at 90 days. The myenteric plexus coordinates intestinal motility by integrative neuronal networks^35^. Neuronal loss (or shrinkage of cell bodies) in the ENS has been described in protozoan infections, often being attributed to inflammation and oxidative stress^33^. However, we observed no marked inflammatory infiltrate, suggesting that additional mechanisms, such as direct parasite-derived factors or neuroimmune dysregulation, may contribute to ENS remodeling in *L. (V.) braziliensis* infection. Given the crucial role of the ENS in coordinating motility and its interactions with the endocrine and immune systems, these findings raise questions about the functional consequences of infection on gastrointestinal physiology.

Taken together, our data show that *L. (V.) braziliensis* infection induces strain- and time-dependent structural and cellular changes in the duodenum. The reference strain MHOM/BR/1975/M2903 and MHOM/BR/2000/1655 (considered resistant) caused more pronounced alterations than others, and changes were more evident at 90 days than at 120 days, possibly reflecting tissue regeneration. The alterations in goblet cells, extracellular matrix organization, and ENS morphology suggest that *Leishmania* infection, although primarily cutaneous, may exert broader systemic effects. These findings highlight the intestine as a potential target organ and hamsters as a valuable experimental model for studying parasite–host interactions and their impact on intestinal physiology.

## CONCLUSION

Infection with distinct *L. (V.) braziliensis* strains induces strain- and timedependent structural and neurochemical alterations in the duodenum of hamsters. Key findings include compromised mucin-secreting goblet cells, extracellular matrix remodeling (increased type III collagen deposition), and disruption of the myenteric ganglion architecture. These results provide new insights into the systemic and intestinal effects of cutaneous *Leishmania* infections, highlighting the relevance of intestinal and enteric nervous system involvement in the pathophysiology of this disease.

## Data Availability

The complete anonymized dataset supporting the findings of this study is included within the article itself.
